# Learning What Not to Do in Community-Engaged Nursing Research in Rural Settings

**DOI:** 10.1093/geroni/igaf122.715

**Published:** 2025-12-31

**Authors:** Lisa Wiese, Magdalena Tolea, Janet Holt

**Affiliations:** Florida Atlantic University, Boca Raton, Florida, United States; University of Miami Miller School of Medicine, Miami, Florida, United States; Florida Atlantic University, Boca Raton, Florida, United States

## Abstract

Although the rural community setting is well-suited for ADRD screening and care management due to trust established between providers and patients, barriers to diagnosis and management reflect a combination of factors. When conducting our “ORCHID” study (Optimizing Rural Community Health to Increase Dementia Detection and Care) to address these barriers, we discovered unique barriers to building capacity for community-engaged and dementia-focused research. Aims in this community and provider education intervention were 1) identify community-specific provider barriers to clinical ADRD assessments, 2) evaluate the effectiveness of pairing local nursing students with faith-based health educators to provide community education and cognitive screening, and 3) compare ADRD diagnosis and treatment outcomes between provider education and control groups. Interventions included support from regional neurology teams, adult gerontology nurse practitioner assessments/recommendations, and training office staff regarding ADRD resources for patients/caregivers. Measures included objective/subjective cognitive assessment surveys for residents, and provider dementia knowledge and confidence surveys. The sample (N = 232 residents;14 providers) was primarily non-White (71.2%), female (72.2%), and living alone (57.5%). Seventy-three (31%) of older adults (age: 65.0±10.1 yrs.) scored at risk for cognitive impairment (Mini-MoCA < 12). The intervention provider group was small (n = 4) but they did increase diagnosis and treatment by 60%, while the control group (n = 10) rates were unchanged. Other findings included determining what was unsuccessful regarding partnering nursing students with community health educators, provider training, referrals, data-gathering events, team composition, timing, and participant stipends. The ORCHID framework revealed important considerations for multidisciplinary approaches to improving ADRD detection and management in rural settings.

